# Low-Dose Quercetin Dephosphorylates AKT and Suppresses Proteins Related to Migration in Human Metastatic Uveal Melanoma Cells

**DOI:** 10.3390/life15060979

**Published:** 2025-06-18

**Authors:** Petra Fodor, József Király, Zsuzsanna Szabó, Katalin Goda, Barbara Zsebik, Gábor Halmos

**Affiliations:** 1 Department of Biopharmacy, Faculty of Pharmacy, University of Debrecen, 4002 Debrecen, Hungary; fodor.petra@pharm.unideb.hu (P.F.); kiraly.jozsef@pharm.unideb.hu (J.K.); szabo.zsuzsanna@pharm.unideb.hu (Z.S.); 2 Doctoral School of Pharmaceutical Sciences, University of Debrecen, 4032 Debrecen, Hungary; 3 Department of Biophysics and Cell Biology, Faculty of Medicine, University of Debrecen, 4032 Debrecen, Hungary; goda@med.unideb.hu

**Keywords:** uveal melanoma, quercetin, metastasis, migration markers, epithelial–mesenchymal transition

## Abstract

**Background:** Uveal melanoma (UM) is the most common intraocular cancer of the eye, with high metastatic potential in adults. In 50% of patients, UM spreads to other tissues, causing a fatal outcome. Flavonoids are bioactive phenolic compounds found in fruits and plants, thus commonly present in the natural diet. Quercetin is the most remarkable agent among flavonols proved to have an anticancer effect. Thus, we aimed to investigate the effect of quercetin on a metastatic UM cell line MM28. **Methods:** MM28 cells were treated with increasing concentrations of quercetin (0.1–10 µM). The changes of proliferation and migration markers were studied both in gene and protein expression level by qPCR, Western blotting, and Proteome Profiler Human XL Oncology Array. **Results:** Quercetin had only a slight anti-proliferative effect on MM28 cells. However, 1 µM of quercetin significantly elevated the mRNA expression of the Maspin gene and downregulated MMP2 gene expression. In addition, the protein expression levels of pAKT, NF-κB, and MMP8 were significantly decreased by the treatment. **Conclusions:** Our findings indicate that low-dose (1 µM) quercetin treatment is able to suppress the expression of certain migration markers, and therefore, it might be a useful adjuvant compound to reduce metastasis formation of UM.

## 1. Introduction

Uveal melanoma (UM) is a rare type of cancer, but the most frequently occurring primary intraocular malignancy. The annual incidence of UM varies greatly in different regions; the age-standardized incidence in Europe is between two and eight cases per million people. In the USA, the age-adjusted incidence is 5.2 cases per million individuals and is 7.2 per million in Australia [1]. Its localization can extend to the choroid (80%), the ciliary body (12%), and the iris (8%) [2,3]. Fair skin, congenital ocular melanocytosis, melanocytoma, and BRCA-1-associated protein 1 (BAP1)-tumor predisposition syndrome are risk factors for its development [2,4]. Opposed to skin melanomas, B-Raf Proto-Oncogene (BRAF) mutations are uncommon in uveal melanoma, while G Protein Subunit Alpha Q (GNAQ) and G Protein Subunit Alpha 11 (GNA11) gene mutations are very frequent [2]. Since this type of tumor shows chemoresistance to applied therapies, the treatment options are limited [2]. In the case of primary tumor, potential treatment options can be radiotherapy, phototherapy, and enucleation for the most advanced cases [2,5]. Approximately 50% of the patients will develop metastasis, mostly in the liver, despite the primary treatment in the following 5 years [2,6]. There is no standard treatment for metastasis, and the used chemotherapeutics are similar to cutaneous melanoma, such as docosahexaenoic acid, dacarbazine, fotemustine, temozolomide, paclitaxel, and liposomal vincristine [2]. Since there is no first-line treatment for this extremely aggressive metastasized tumor, there is an urgent need for new treatment protocols.

Quercetin is a flavonoid derived from plants and fruits that is naturally present in the diet. It has been shown to have an anti-tumor effect against different types of cancer, such as leukemia [7], liver [8], gastric [9], lung [10], prostate [11], colon [12], and breast [13] cancers. Due to its antiproliferative effect on tumor cells [14] and its well-known inhibitory effect on ATP-binding cassette (ABC) transporters related to multidrug resistance [15], it can function as a suitable agent for controlling metastasis when added as an adjuvant agent to cytotoxic drugs such as doxorubicin [16].

In many studies, the antitumor activity of quercetin was observed at high concentrations, between 20 μM and 200 μM. However, according to pharmacokinetic studies, the achievable peak concentration of quercetin in blood is 10 μM [14]. Therefore, we aimed to investigate the effects of low-dose quercetin treatment (0.1–10 μM) applied for 24 h to 72 h duration to metastatic uveal melanoma cells.

The phosphatidylinositol 3-kinase/protein kinase B (PI3K/AKT) signaling transduction pathway is often over-activated in various types of cancer. It plays a key role in the proliferation, migration, invasion, and metastasis of tumor cells [17]. Various studies have showed that quercetin has an effect on the PI3K/AKT pathway and it can regulate the ability of tumor cells to invade and migrate by downregulating the phosphorylation of AKT [18,19,20,21].

The PI3K/AKT pathway also has a major role in matrix metalloproteinase (MMP)-mediated tumor cell migration and invasion in various types of cancer. MMP family members, such as MMP2, MMP8, and MMP9, have important roles in degrading the extracellular matrix (ECM), contributing to migration and metastasis formation in various cancers [21]. Quercetin was shown to suppress cell migration and invasion in glioblastoma [22], melanoma [23], and cancer cells of oral [24], pancreatic [25], and breast cancers [26] by decreasing MMP levels.

Activation of nuclear factor-kappaB (NF-κB) can be induced by different signaling pathways, such as PI3K/AKT [27]. This transcription factor can regulate proliferation, migration, and cell differentiation [28]. Previous studies demonstrated that quercetin treatment caused NF-κB protein depletion [29,30,31]. Epithelial–mesenchymal transition (EMT) is also involved in migration, tumor progression, resistance to cancer treatment, and formation of metastasis [32,33]. Since EMT is only partially finished in tumor cells, they express both epithelial and mesenchymal genes. This mixed morphology in tumorous cells can cause a more aggressive phenotype compared to normal cells [34]. The most common cases of UM represent a mixed epitheloid–spindle cell type (48%), followed by spindle-B cell tumors (32%). This heterogeneity may be the cause of chemoresistance, thus serving as a major obstacle in therapy [35].

Many findings stated that quercetin has an inhibitory effect on EMT in different ways. For instance, in a human papillary thyroid cancer cell line, quercetin induced the proteolysis of vimentin [36]. On the other hand, in oral squamous cell carcinoma, quercetin upregulated the expression of epithelial markers, such as E-cadherin, while downregulating mesenchymal markers like vimentin [37]. Xinxing Lu et al. (2020) found that quercetin downregulates the expression of Metastasis-Associated Lung Adenocarcinoma Transcript 1 (MALAT1) long non-coding mRNA, which results in inhibition of EMT in prostate cancer cells [38].

In our study, our aim was to investigate the antitumor effect of low-dose quercetin on a metastatic uveal melanoma cell line MM28, regarding changes in the gene and protein expression levels of proliferation and migration markers. Moreover, as knowledge and research on uveal melanoma is relatively limited, we aimed to investigate EMT in the MM28 UM cell line in response to 1 µM quercetin treatment.

## 2. Materials and Methods

### 2.1. Cell Line and Culturing Conditions

The MM28 human metastatic uveal melanoma cell line was purchased from ATCC (CRL-3295). Cells were cultured in complete growth medium RPMI-1640 (30-2001) containing 2 mM L-glutamine, 10 mM HEPES, 1 mM sodium pyruvate, 4500 mg/L glucose, and 1500 mg/L sodium bicarbonate, supplemented with 20% fetal bovine serum (FBS), 100 U/mL penicillin, and 100 mg/mL streptomycin in a humidified chamber (95% air humidity, 5% CO_2_) at 37 °C.

### 2.2. Reagents and Cell Viability Determination by MTT Assay

Quercetin was purchased from SIGMA (Q4951), dissolved in DMSO as a vehicle, and stored as a stock solution in aliquots at −20 °C. In each experiment, control samples were treated with the amount of DMSO used to dilute the applied concentration of quercetin (0.1–10 µM).

MTT (3-(4,5-dimethylthiazol-2-yl)-2,5-diphenyltetrazolium bromide) tetrazolium assay (St. Louis, MO, USA) was used to determine cell viability. Cells were seeded into a 96-well plate at the density of 6000 cells/well in complete growth media and incubated for 24 h. Each well contained 200 µL medium. The next day, the entire media was replaced with culture medium containing increasing concentrations of quercetin (0.1–10 µM). All types of treatments were performed in triplicate (*n* = 3). The cells were treated for 24–72 h with fresh media combined with quercetin being added every 24 h and were maintained at 37 °C in the humidified chamber with 5% CO_2_/95% air humidity. The viability of cells was measured every 24 h by adding the MTT reagent to the media; the plates were incubated for 2 h at 37 °C in the cell culture incubator. Afterwards, the media was aspirated from the wells, replaced by 100 µL DMSO, and incubated for 15 min at 37 °C. Absorbance was measured at 560 nm, with 660 nm as reference in a FLUOstar Optima Counter (BMG Labtech GmbH, Ortenberg, Germany). Values were expressed relative to the control. Doxorubicin in a concentration range of 0.1–10 µM (2 mg/mL injection solution, TEVA, Debrecen, Hungary) was used as a positive control due to its significant role in inducing cell death.

### 2.3. RNA Isolation

To investigate the changes in the expression of genes involved in proliferation and migration (AKT, PI3K, PTEN, MMP2, MMP9, Maspin, Endoglin, HO-1, Vimentin; Integrated DNA Technologies, Coralville, IA, USA), MM28 cells were treated with 1 µM of quercetin for 72 h, and then total RNA was isolated. Treatments were performed in biological replicates (*n* = 3). For total RNA extraction, the NucleoSpin RNA/Protein kit (Macherey-Nagel, Düren, Germany) was used according to the manufacturer’s protocol.

### 2.4. Quantitative Real-Time PCR (qRT-PCR) Analysis

RNA was reverse-transcribed to cDNA from 1000 ng of total RNA by using the Tetro cDNA Synthesis Kit (Bioline, London, UK). In order to investigate the expression of genes involved in proliferation and migration after treatments, the qRT-PCR method was used. A total of 100 ng of reverse-transcribed cDNA was applied for qRT-PCR, carried out with specific primers (Supplementary Table S1), using GAPDH as a housekeeping gene. qRT-PCR arrays were performed with iQ™ SYBR^®^ Green Supermix (Bio-Rad Laboratories, Hercules, CA, USA) according to the manufacturer’s protocol using a CFX-96 Real-Time System (BioRad Laboratories Inc., Hercules, CA, USA) in 10 µL final reaction volume in biological replicates (*n* = 3). Samples were subjected to an initial annealing step (95 °C for 3 min), followed by 40 amplification cycles (95 °C for 15 s, optimal melting temperature for each primer for 1 min) and 81 cycles of melting (55–98 °C for 0.5 min/°C), respectively. Gene expression has been quantified by the ΔΔCt method. Results were normalized to the expression of the GAPDH gene.

### 2.5. Protein Isolation

The MM28 cells were treated with 1 μM quercetin for 72 h. After the treatment, the cells were rinsed with PBS and lysed in Lysis Buffer 17 (R&D Systems, Inc., Minneapolis, MN, USA), supplemented by 10 µg/mL Aprotinin, 10 µg/mL Leupeptin, 10 µg/mL Pepstatin protease inhibitors, and Phosphatase Inhibitor Cocktail 2 (Sigma-Aldrich, St. Louis, MO, USA) in a ratio of 1:100. Protein quantification was performed by the Bradford assay.

### 2.6. Western Blot

Samples were diluted with 4×Laemmli buffer in dH_2_O and boiled at 95 °C for 5 min. A total of 30 µg protein was separated on 10% SDS PAGE and transferred to the PVDF membrane. We used 5% non-fat dry milk as a blocking agent in Tris-buffered saline containing 0.1% Tween-20. Specific antibodies were used to detect the proteins listed in Supplementary Table S2. All of the primary antibodies were applied in a 1:1000 concentration. Primary antibodies were labeled with horseradish peroxidase (HRP)-tagged anti-rabbit IgG secondary antibody (Thermo Fisher Scientific, Waltham, MA, USA) and detected using Clarity Western ECL Substrate (Bio-Rad Laboratories, Hercules, CA, USA). Results were acquired and analyzed with the ChemiDoc Imaging System using Image Lab Software 5.2 (Bio-Rad Laboratories, Hercules, CA, USA). Samples were normalized to Hypoxanthine Phosphoribosyltransferase (HPRT) (Sigma-Aldrich, St. Lous, MO, USA). Samples were run under identical loading conditions (100 V, 90 min).

### 2.7. Proteome Profiler Human XL Oncology Array

Cell lysates were analyzed for 84 oncogenes using the Proteome Profiler Human XL Oncology Array kit (R&D systems, Minneapolis, MN, USA) according to the manufacturer’s protocol. A total of 150 µg of protein extract was used for each membrane in Human XL Oncology Array analysis. The protein extracts were mixed with Array Buffer 4 and Array Buffer 6, if necessary, to adjust to a final volume of 1.5 mL. The membranes were blocked by Array Buffer 6 for one hour at a rocking platform shaker. The buffer was aspirated from the 4-Well Multi dish, and then the sample mixture was added to the membranes and they were incubated overnight at 4 °C. After the overnight incubation, the membranes were washed with 1× Wash Buffer three times, then incubated with Detection Antibody Cocktail diluted in Array Buffer 4/6 at room temperature for 1 h on a rocking platform shaker. Afterwards, the membranes were washed with 1× Washing Buffer three times. Then, 2 mL of 1× Streptavidin-HRP was pipetted onto each array, and the mixture was incubated for 30 min at room temperature, followed by three washes of the membranes. Following the washes, 1 mL of Chemi Reagent Mix was pipetted evenly onto each membrane. After 1 min of incubation, the protein duplicates were detected by a ChemiDoc Imaging System (Bio-Rad, Hercules, CA, USA). The densitometric analysis of the protein arrays was performed using Image Lab Software 5.2 (Bio-Rad Laboratories, Hercules, CA, USA), and the average intensity was determined by subtracting the average background signal [39].

### 2.8. Statistical Analysis

The calculations of mean values, normalization, and standard deviation were performed by Microsoft Excel (Office Professional Plus 2016, Microsoft, Redmond, WA, USA). Statistical significances were calculated by Students’s *t*-test or one-way ANOVA test in GraphPad Prism 8 software (GraphPad Software Inc., San Diego, CA, USA). A *p* value * < 0.05 was considered to be statistically significant (*p* value ** < 0.005: very significant, *p* value *** < 0.0005: highly significant, *p* value **** < 0.0001: extremely significant).

## 3. Results

### 3.1. Effect of Quercetin on the Cell Viability of MM28 Metastatic UM Cells

MM28 metastatic uveal melanoma cell line was used to investigate the effect of low-dose quercetin on cell viability. Cells were treated with different concentrations of quercetin (0.1, 1, and 10 µM), and cell viability was analyzed by MTT-assay at different time points.

**Figure 1:**
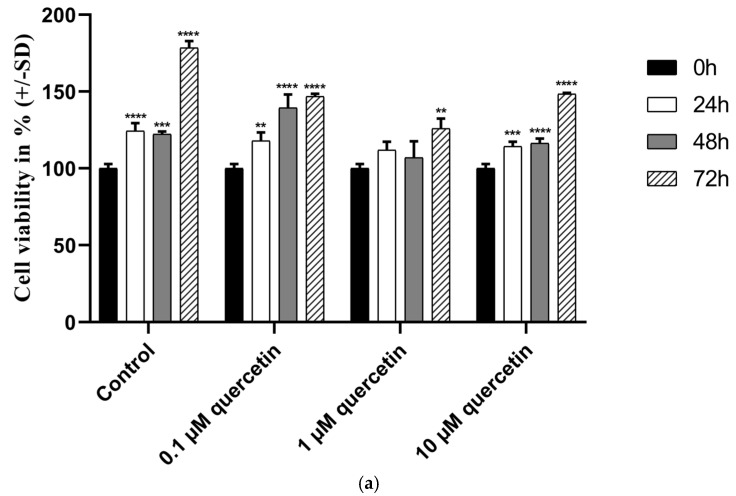

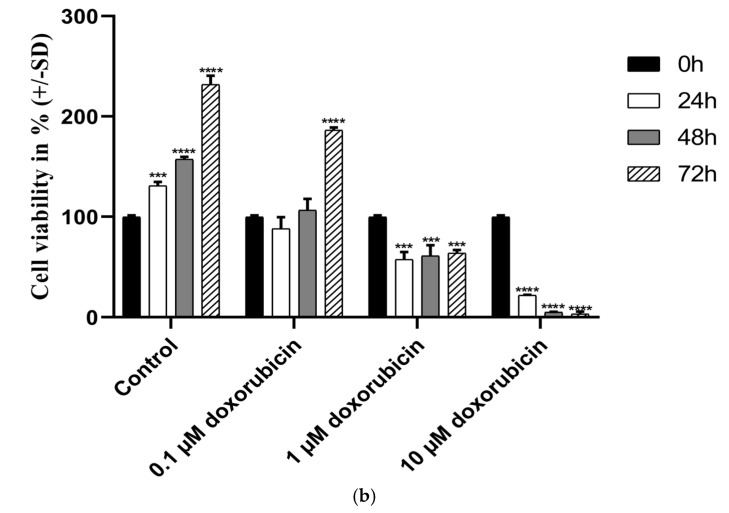
Cell viability of MM28 human metastatic uveal melanoma cells (**a**) treated with 0–10 µM of quercetin (**b**) and 0.1–10 µM of doxorubicin, which serves as a positive control. The absorbance of treated samples was normalized to untreated control supplemented with DMSO at 0 h. One-way ANOVA test was applied for statistical analysis (** *p* < 0.005, *** *p* < 0.0005, **** *p* < 0.0001).

According to our MTT assay, compared to the control at different time points, 1 μM of quercetin did not significantly reduce the cell viability at 24 h (*p* = 0.0546) and 48 h (*p* = 0.0709), although at 72 h, the treatment caused a significant reduction (*p* = 0.0003). Surprisingly, a comparison between the 1 µM and 10 µM quercetin treatments at 72 h revealed a significant increase (*p* = 0.0039) in viability at a higher concentration (Figure 1a). Therefore, we further studied whether 1 µM of quercetin at 72 h, with an anti-proliferative effect, could modify the expression of proteins involved in metastasis formation. As doxorubicin is known to promote cell death, it was used as a positive control (Figure 1b). Our results showed that doxorubicin at concentrations of 1 and 10 µM demonstrated significant inhibition of cell viability, even after 24 h (*p* = 0.0002, *p* < 0.0001). However, 1 µM of quercetin showed a statistically significant effect only after 72 h.

### 3.2. Effect of Quercetin on Genes Related to Proliferation and Migration of MM28 Metastatic UM Cells

The PI3K/AKT signaling transduction pathway has a crucial role in various types of cancer. It regulates important factors of cell viability, angiogenesis, and metabolism. Also, it plays important roles in cell proliferation and migration [40]. Quercetin has been reported in many studies to control the PI3K/AKT signaling transduction pathway [19,20,21]. Therefore, we hypothesized that it would be worthwhile to examine what changes occur in this pathway after low-dose quercetin treatment, even if a high degree of inhibition of cell survival was not observed in this case.

**Figure 2:**
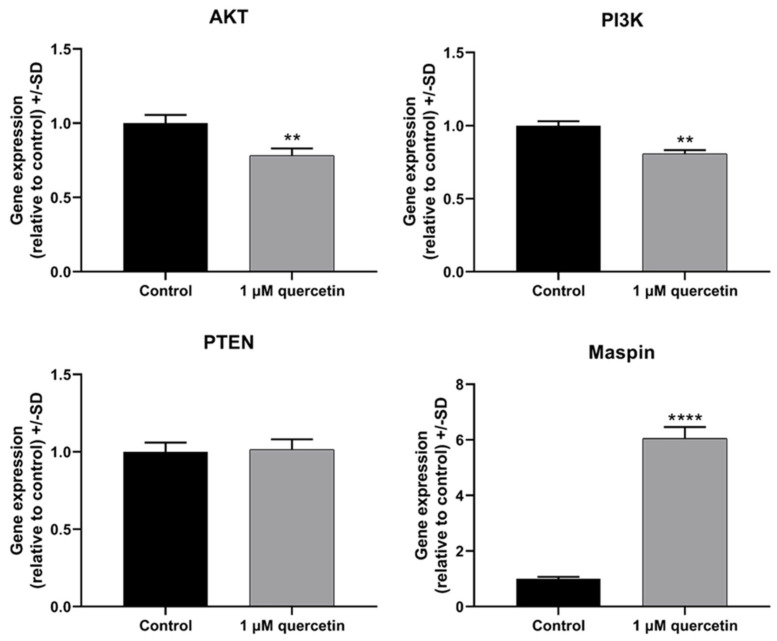
mRNA expression levels of proliferation markers in MM28 cells after 1 µM quercetin treatment for a 72 h period. Total RNA was isolated, and then 100 ng of cDNA was used for investigation of the gene expression of AKT, PI3K, PTEN, and Maspin, using GAPDH as a housekeeping gene. The expression levels in samples treated by quercetin were normalized to control samples (untreated). Treatments were performed in three independent experiments and were expressed as the mean ± S.D. Student’s *t*-test was applied for statistical analysis (** *p* < 0.005, **** *p* < 0.0001). After the treatment, there was no significant change in PTEN (*p* = 0.7711), whereas AKT (*p* = 0.0047) and PI3K (*p* = 0.0011) significantly decreased, and Maspin (*p* < 0.0001) showed a significant elevation.

**Figure 3:**
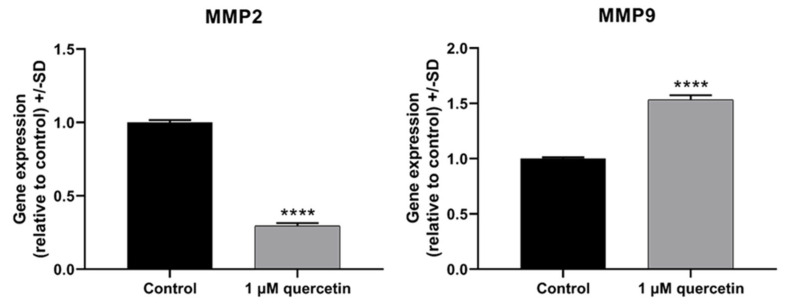
mRNA expression levels of migration markers in MM28 cells after 1 µM quercetin treatment for a 72 h period. Total RNA was isolated, then 100 ng of cDNA was used for the analysis of the expression of MMP2 and MMP9 genes, using GAPDH as a housekeeping gene. The expression levels of quercetin-treated samples were normalized to untreated control samples. Treatments were performed in three independent experiments and were expressed as the mean ± S.D. Student’s *t*-test was used for statistical analysis (**** *p* < 0.0001). After the treatment, there were opposite significant changes in MMP2 (*p* < 0.0001) and MMP9 (*p* < 0.0001).

In the first experiment, the mRNA expression of PI3K, AKT, Phosphatase and Tensin Homolog (PTEN), and Maspin genes were examined in MM28 cells by qRT-PCR after the treatment with 1 µM of quercetin for 72 h. It was observed that the quercetin treatment significantly reduced the gene expression of AKT and PI3K and increased the gene expression of the transcription factor Maspin, while there was no change in PTEN (Figure 2). Since the PI3K/AKT pathway also plays a pivotal role in MMP2/MMP9-mediated tumor cell migration and invasion in various types of cancer, in the following experiments, we studied the effects of low-dose quercetin on the mRNA expression of proteins that participate in cell signaling pathways that affect the formation of metastases. MMP2 and MMP9 are members of the MMP family and play a significant role in ECM degradation, thus promoting the migration of tumor cells and their ability to metastasize [21]. Figure 3 shows that quercetin caused a significant decrease in MMP2, while increasing MMP9.

### 3.3. Dephosphorylation Effect of Quercetin on AKT Protein in MM28 Cells

**Figure 4:**
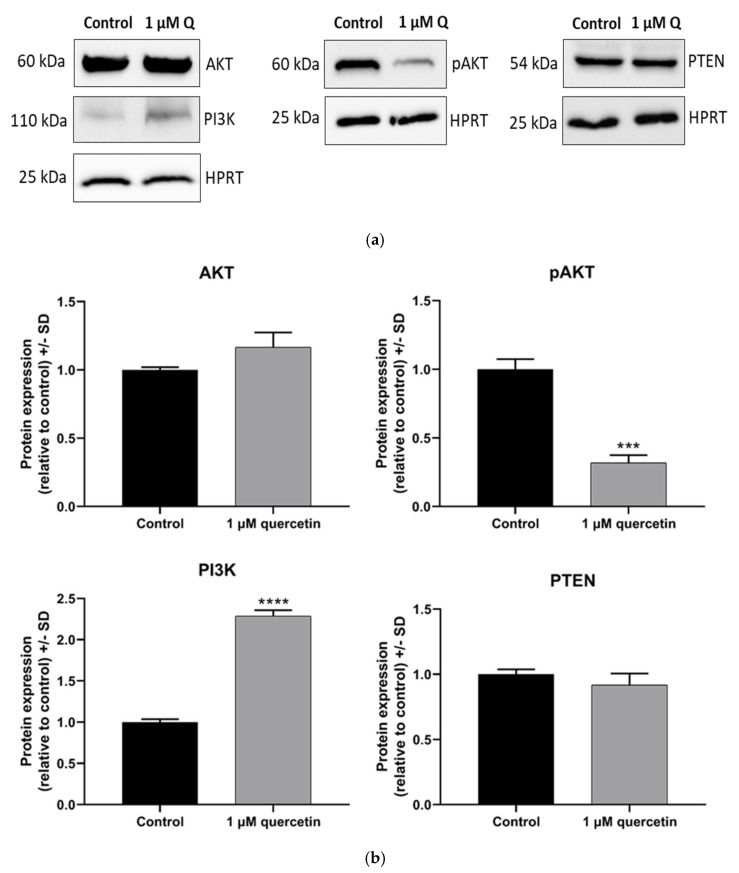
Western blot analysis of proliferation markers in MM28 cells after 1 µM of quercetin treatment for a 72 h period. The protein expression levels of quercetin-treated samples were normalized to untreated control samples. (**a**) Western blot images of AKT, pAKT, PI3K, PTEN, and HPRT proteins. (**b**) Band intensities of AKT, pAKT, PI3K, and PTEN protein expressions. The protein expressions were normalized to the HPRT housekeeping protein. Treatments were performed in three independent experiments and were expressed as the mean ± S.D. Student’s *t*-test was used for statistical analysis (*** *p* < 0.0005, **** *p* < 0.0001). After the treatment, there were no significant changes in AKT (*p* = 0.0576) and PTEN (*p* = 0.3481), while pAKT (*p* = 0.0002) and PI3K (*p* < 0.0001) showed opposite significant changes.

In the following experiments, we studied the effects of 72 h low-dose quercetin treatment on the expression of proteins involved in the PI3K/AKT pathway, focusing on PI3K, AKT, phosphorylated-AKT (pAKT), and PTEN. According to Western blot analysis (Figure 4), the expression level of PI3K increased by twofold, while the expression levels of AKT and PTEN did not change significantly. Interestingly, a strong decrease in pAKT protein level was observed (Figure 4).

### 3.4. Effect of Quercetin on the Expression of NF-κB Protein

**Figure 5:**
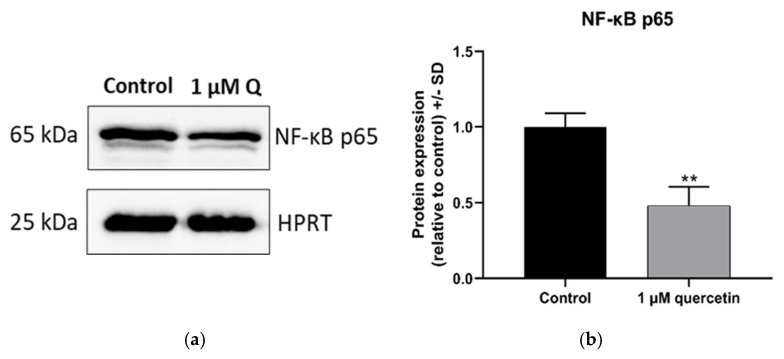
Changes in protein expression level of NF-κB p65 in MM28 cells by Western blot analysis after 1 µM quercetin treatment for a 72 h period. The expression levels of quercetin-treated samples were normalized to control samples (untreated). (**a**) Western blot images of NF-κB p65 and HPRT proteins. (**b**) Band intensities of NF-κB p65 protein expression. Treatments were performed in three independent experiments and were expressed as the mean ± S.D. Student’s *t*-test was used for statistical analysis (** *p* < 0.005). After the treatment, there was a significant decrease in NF-κB p65 (*p* = 0.0043).

The NF-κB protein has a pivotal role in signaling pathways regulating cell migration and proliferation. The NF-κB family involves many transcription factors, including a subunit, p65, that contains a transactivation domain, which interacts with the transcriptional apparatus [27]. The 1 µM quercetin treatment caused significant reduction in NF-κB subunit p65 protein levels (Figure 5).

### 3.5. Effect of Quercetin on Matrix Metalloproteinase Proteins

**Figure 6:**
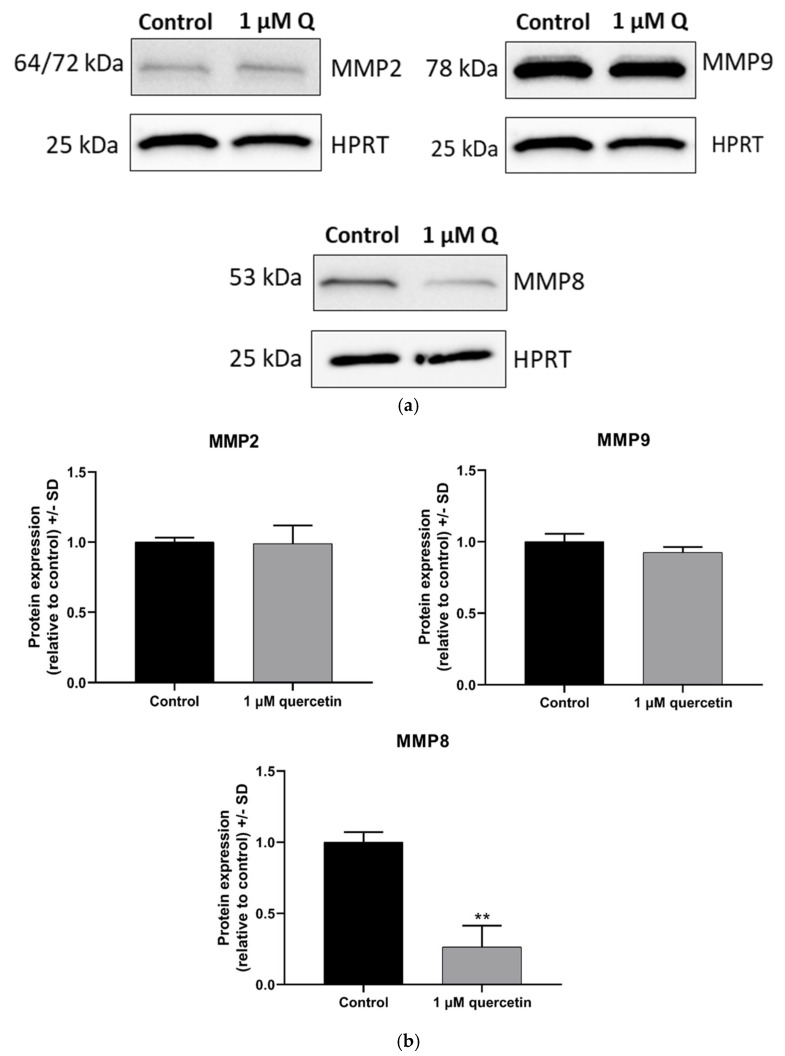
Western blot analysis of protein expression levels of MMP2, MMP8, and MMP9 in MM28 cells after 1 µM of quercetin treatment for a 72 h period. The expression levels of quercetin-treated samples were normalized to untreated control samples. (**a**) Western blot images of MMP2, MMP8, MMP9, and HPRT proteins. The PVDF membrane probed with an antibody for pAKT (Figure 4) was stripped and reprobed with an antibody for MMP8; using the same HPRT band for densitometric analysis, samples were running under identical loading conditions. (**b**) Band intensities of MMP2, MMP8, and MMP9 protein expressions. Treatments were performed in three independent experiments and were expressed as the mean ± S.D. Student’s *t*-test was used for statistical analysis (** *p* < 0.005). After the treatment, there were no significant changes in MMP2 (*p* = 0.9473) and MMP9 (*p* = 0.1273), while MMP8 was greatly suppressed (*p* = 0.0027).

We also studied the effects of quercetin on the expression of matrix metalloproteinase enzymes by Western blot. Besides MMP2 and MMP9 studied in qRT-PCR experiments, we also included MMP8, since a recent study by Dan-Ning Hu et al. (2018) observed high constitutive levels of MMP8 in uveal melanocytes [41]. Another study suggested that MMP8 plays dual roles by promoting or suppressing cancer progression depending on the type of cancer [42]. According to Gutierrez et al. (2008), MMP8 prevents metastasis formation through modifying the adhesion and invasion of the tumorous cells [43]. In our experiments, 1 µM quercetin treatment did not change the MMP2 protein expression, and the extent of MMP9 reduction was not statistically significant (*p* = 0.1273); however, the MMP8 enzyme (not analyzed by qRT-PCR) showed a significant decrease (Figure 6).

### 3.6. Effect of Quercetin Treatment on Proteins Related to EMT

Numerous findings proved that quercetin has many effects of EMT regulating markers and factors, although the number of publications about EMT in human metastatic uveal melanoma are very limited [37,38,44]. Thus, we intended to apply a screening technique to obtain a broader perspective about the effect of quercetin on EMT-regulating factors. Using Human XL Oncology Array (R&D), we were able to study these markers efficiently.

**Figure 7:**
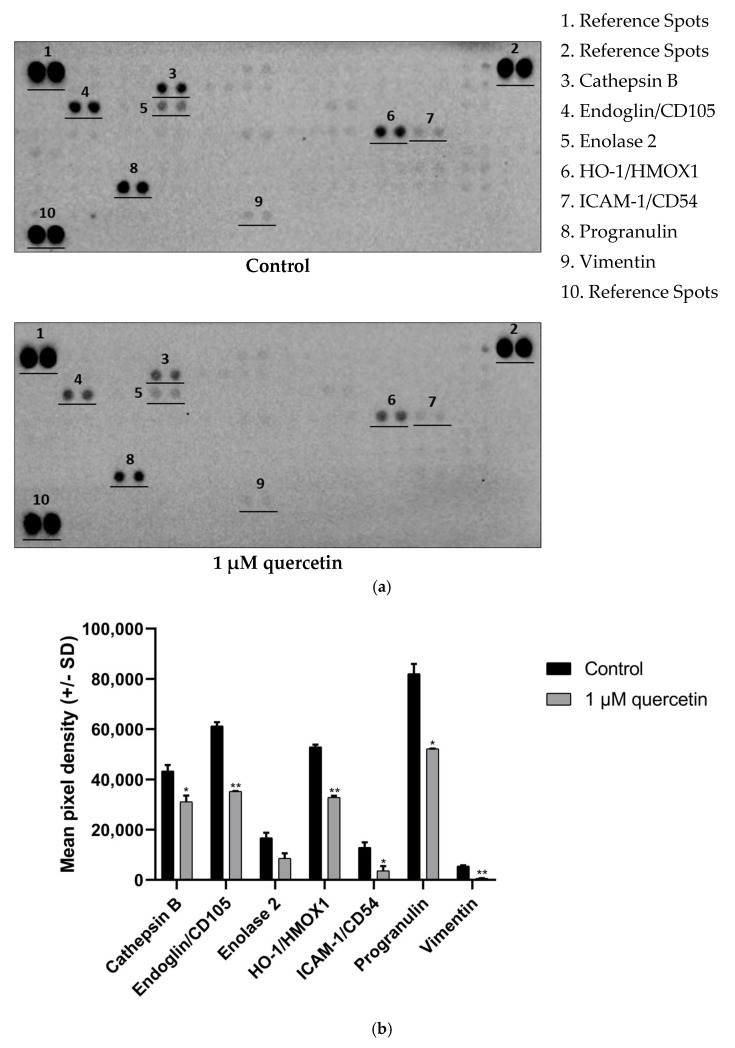
Proteome Profiler Human XL Oncology Array analysis of Cathepsin B, Endoglin/CD105, Enolase 2, HO-1/HMOX-1, ICAM-1/CD54, progranulin, and vimentin protein expression levels in MM28 cells after 1 µM of quercetin treatment for a 72 h period. (**a**) Oncology array pictures of control and 1 µM quercetin treated samples of MM28 cells. (**b**) Mean pixel density of Cathepsin B, Endoglin/CD105, Enolase 2, HO-1/HMOX-1, ICAM-1/CD54, progranulin, and vimentin after treatment with 1 µM quercetin. The expression levels of quercetin-treated samples were normalized to untreated control samples. Data represent mean values ± S.D. Student’s *t*-test was used for statistical analysis (* *p* < 0.05, ** *p* < 0.005). After the treatment, there was no significant change in Enolase 2 (*p* = 0.0539), while Cathepsin B (*p* = 0.0371), Endoglin/CD105 (*p* = 0.0016), HO-1 (*p* = 0.0016), ICAM-1 (*p* = 0.0414), progranulin (*p* = 0.0083), and vimentin (*p* = 0.0028) showed a significant reduction.

The 1 µM quercetin treatment altered the expression pattern of EMT markers. Interestingly, a large-scale significant reduction was detected in one of the main EMT markers, vimentin. We also observed strong downregulation of Endoglin and Heme Oxygenase 1 (HO-1). Other proteins detected, namely, intercellular adhesion molecule 1 (ICAM-1), Cathepsin B, and progranulin, also showed significant decrease in our array (Figure 7).

### 3.7. Effect of Quercetin Treatment on Genes Related to EMT

**Figure 8:**
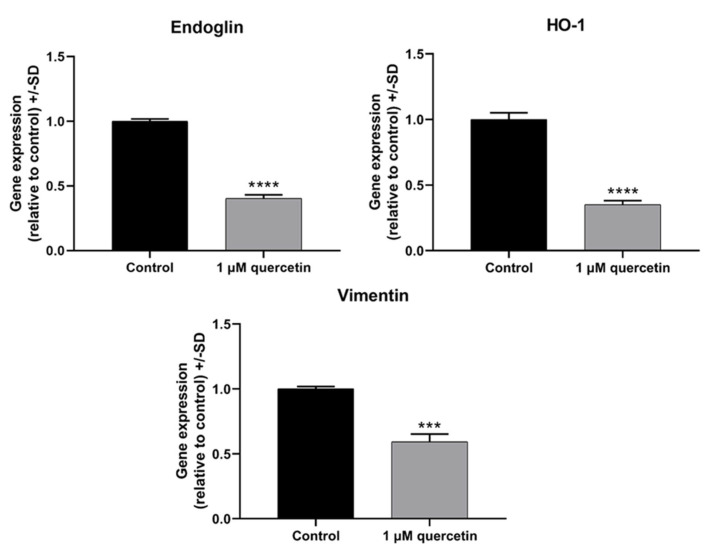
mRNA expression levels of Endoglin, HO-1, and vimentin in MM28 cells after 1 µM quercetin treatment for a 72 h period. Total RNA was isolated, then 100 ng of cDNA was used for the evaluation, using GAPDH as a housekeeping gene. The expression levels of quercetin-treated samples were normalized to untreated control samples. Treatments were performed in three independent experiments and were expressed as the mean ± S.D. Student’s *t*-test was applied for statistical analysis (*** *p* < 0.0005, **** *p* < 0.0001). After the treatment, there were significant reductions in Endoglin (*p* < 0.0001), HO-1 (*p* < 0.0001), and vimentin (*p* = 0.0003).

To analyze whether the protein expression profile changes detected by the Proteome Profiler Human XL Oncology Arrays also existed on the level of mRNA expression, we also performed real-time qRT-PCR experiments on the markers that showed highly significant reduction. The control and quercetin-treated samples were analyzed with gene-specific primers for Endoglin, HO-1, and vimentin. Our qRT-PCR experiments demonstrated that all of the investigated markers were significantly downregulated in response to 1 µM quercetin treatment (Figure 8).

## 4. Discussion

Uveal melanoma is an intraocular malignancy, originating from extracutaneous melanocytes residing within the uveal tract of the eye. Despite successful therapy of the primary tumor, 40–50% of patients develop distant metastases, most commonly in the liver (93%). First-line treatments for the primary tumor are mainly localized, although for the metastasized tumor, there is no routine protocol, and individuals may get targeted therapies, checkpoint inhibitors, or antibody treatment with a patient-specific approach [1,45]. The lack of effective treatment is an urgent problem, and it encourages new agents to be developed for the treatment of this aggressive type of cancer. A natural flavonoid, quercetin shows antitumor effects in various cancer models [7,10,13]. In uveal melanoma, there is very limited information available about the antitumor effect of this specific polyphenol compound [46,47].

In our study, we aimed to investigate markers affecting proliferation and migration by quercetin treatment in human metastatic UM cells. After 72 h treatment with 1 μM of quercetin, the applied concentration significantly reduced the cell viability compared to the untreated control, whereas after 24 h and 48 h, the treatment was not significantly effective. Unexpectedly, a comparison between the 1 µM and 10 µM quercetin treatments at 72 h showed a significant increase in viability at the higher concentration. In a previous study, it was stated that low-dose quercetin does not have the same effect on different breast cancer cell lines. Jeong et al. (2009) reported that the cell viability of MCF-10A was increased at 72 h after a 5 µM of quercetin treatment [14]. In addition to our results with quercetin, doxorubicin was also investigated in our study as a commonly used chemotherapeutic agent to compare the effect of quercetin and doxorubicin on cell viability. Based on our results, doxorubicin in 1 and 10 µM concentrations showed significant inhibition on cell viability, even after 24 h. However, 1 µM of quercetin showed a statistically significant effect only after 72 h. Similarly to our results, a previous study investigating various tea and leaf extracts from different plants also showed a strong antiproliferative effect of doxorubicin used as a positive control [48]. In another study, *Haberlea rhodopensis* extracts were used to determine their cytotoxic effects on various cancer cell lines, and doxorubicin was also used as a reference compound. This study demonstrated that doxorubicin had a greater cytotoxic effect than the investigated M2 plant extract on cancer cells [49]. Quercetin slightly affected the expression of PTEN on mRNA and protein levels, but interestingly, the expression of PI3K protein was increased by twofold. The significant downregulation of PI3K mRNA, in contrast to a significant increase in protein expression, is a puzzling situation, although quercetin is known to cause various epigenetic modifications [50]. As was demonstrated in an earlier study with rhabdomyosarcoma cells, PANX1 mRNA was decreased, but PANX1 protein showed a significantly increased level by quercetin treatment [51]. In parallel, the phosphorylation of AKT was significantly decreased by quercetin treatment, similarly to other studies in various types of cancers published before [52,53].

The treatment of quercetin was also associated with a significant decrease in NF-κB protein expression levels, similarly to previous findings [54,55]. An earlier study stated that NF-κB inhibition reduces MMP levels [56], and therefore in our study, we decided to evaluate the protein expression of MMP2, MMP8, and MMP9 after treatment with 1 µM quercetin. MMP2 and MMP9 are known to induce the dissemination of tumor cells [57]. El Shabrawi et al. (2001) have found that patients with UM tumors that express high levels of MMP2 and MMP9 have poor clinical outcome [58]. In our findings, MMP2 was not altered, and the decline in MMP9 protein levels was not statistically significant, whereas MMP8 protein showed significant decrease as a result of 1 µM quercetin treatment. It was also reported that quercetin in higher doses than 1 µM significantly inhibited MMP9 in a flavonoid–enzyme interaction model and in rats with diabetic retinopathy [59,60]. The significant downregulation of mRNA for MMP2, while the protein expression of MMP2 is not altered, can be a somewhat unexpected result. The cause of this expression difference could be a similar regulatory mechanism, as seen in PI3K expression level changes.

Interestingly, we observed a significant upregulation of MMP9 mRNA levels following 1 µM quercetin treatment, despite the result in MMP9 protein expression. The upregulation of mRNA for MMP9 is unexpected, given its well-known role in promoting metastasis. This discrepancy suggests that quercetin can modulate markers on a post-transcriptional level too, like regulating miRNAs (specially miR-21 and let-7 family) [61]. A study by Wang et al. (2024) investigated the effects of quercetin on lipopolysaccharide (LPS)-treated HTR-8/SVneo trophoblast cells. The study found that treatment with concentrations of 1, 5, and 10 μM quercetin led to a significant increase in MMP9 mRNA levels. While quercetin is often reported to downregulate MMP9 in various cancer models, this study demonstrated that under certain inflammatory conditions, quercetin might have an opposite effect [62].

These changes suggest the potential inhibition of EMT; therefore, we intended to monitor some of the regulators related to EMT by Proteome Profiler Human XL Oncology Array. Cathepsin B is a cysteine protease involved in tumor progression and represents a potential therapeutic target in various human cancers and is also known to play a key role in invasion and migration in tumor cells. For instance, it intensifies the expression of the MMPs by inactivating their inhibitors in human articular chondrocytes, resulting in high level of MMPs, hence fostering ECM degradation [63]. There are also studies demonstrating that quercetin downregulates cathepsin B in cervical cancer cells [64] and in aged Institute of Cancer Research (ICR) mice [65]. Similarly, we observed a significant decrease in cathepsin B expression in response to 1 µM quercetin treatment in MM28 UM cells. Simultaneously, we detected a significant decrease in MMP8 and a slight decrease in MMP9 protein expressions by Western blot and a significant decrease in MMP2 gene expression by qPCR array. A similar observation of this effect was reported by Vijayababu et al. (2006) [66] and Chuang et al. (2016) [67].

Vascular mimicry is a process where aggressive tumor cells form vascular-like channels independent of endothelial cells, contributing to tumor growth, metastasis, and poor clinical outcomes [68]. This progress in uveal melanoma is associated with poor survival rate in patients [69]. Tumors bearing this aberrant blood supply system express endothelial cell markers, such as Endoglin [70]. Junhui Hu et al. (2018) demonstrated that Endoglin/CD105(+) cells in clear cell renal cell carcinoma (ccRCC) have higher motility [41]. In our experiments, 1 µM of quercetin treatment caused a significant reduction in Endoglin/CD105 expression in MM28 cells. To the best of our knowledge, this is the first report about the inhibitory effect of quercetin on Endoglin expression.

In addition, Zheng et al. (2020) demonstrated that glycolytic enzyme Enolase 2 (ENO2) can induce EMT in patients with pancreatic ductal adenocarcinoma (PDAC), thus developing metastasis of PDAC cells [71]. According to our results, the expression level Enolase 2 was reduced by half after 1 µM quercetin treatment. As Leiherer et al. (2016) had stated before, quercetin can downregulate ENO2 [72].

The protection against reactive oxygen species (ROS) requires many antioxidant proteins, such as HO-1. This enzyme is overexpressed in case of stress and oxidative damage. Also, it can reduce inflammatory responses and rate of apoptosis [73]. An earlier study demonstrated that HO-1 was able to increase cancer cell proliferation, migration, and invasion in HEK293T, HeLa, H1299, A549, and DU145 cells in vitro [74]. According to our results, the quercetin treatment significantly lowered the expression of HO-1, similarly to another study that found the same effect [75], possibly weakening the defense against ROS and inhibiting migration of MM28 cells.

Intercellular Adhesion Molecule-1 (ICAM-1) has a substantial role in cell proliferation and motility. In triple negative breast cancer, ICAM-1 has been shown to promote EMT through different pathways [76]. As shown in Figure 7b, 1 µM quercetin treatment caused a significant reduction in ICAM-1 protein expression, possibly decreasing cell motility. Another study published previously has stated that quercetin can decrease the protein expression level of ICAM-1 in epithelial cells [77].

Progranulin is known to facilitate invasion and migration of cancer cells through various mechanism of actions. In ovarian cancer cell line A2780, progranulin regulated the EMT-process through increased vimentin and Twist Family BHLH Transcription Factor 1 (Twist) expressions and also decreased the levels of E-cadherin and cytokeratin, thus promoting cell invasion and migration [78]. In our study, 1 µM quercetin had an inhibitory effect on progranulin expression after 72 h treatment. However, changes in the expression of progranulin have not yet been investigated after treatment with quercetin in other studies.

In cancer metastasis, vimentin is permanently overexpressed in cancer cells, defending them from stress and supporting cell organelles during cancer progression and EMT [79]. Wen-wei Chang et al. (2012) suggested that quercetin can decrease the migration ability of head and neck cancer cells through downregulation of vimentin [80]. In our study, we found a similar decrease in vimentin expression after 1 µM quercetin treatment by Proteome Profiler Human XL Oncology Array, and the reduction of endoglin, HO-1, and vimentin was also observed by qRT-PCR. Importantly, the downregulation of Endoglin, HO-1, and vimentin observed in the protein array was in agreement with the mRNA expression data (Figure 8), further supporting our findings.

Some limitation of our study is the use of a single cell line (MM28), which may not fully represent the heterogeneity of UM tumors. In the near future, based on our ongoing investigation using additional cell line(s), we could provide broader conclusions to clarify further questions.

In conclusion, low-dose quercetin treatment can significantly reduce the expression of cell migration markers in MM28 UM cells, while it has only a moderate effect on cell proliferation. The suppressing effect of quercetin on migration markers raises the possibilities that this flavonoid can be applied as an adjuvant compound together with cytotoxic drugs for the treatment of human metastatic UM. Analogously, quercetin potentiated the cytotoxic effect of doxorubicin in breast cancer cells by reducing the expression of ABC transporters, while diminishing its toxic effects in normal cells [16]. Based on these results, quercetin could function as a valuable and potent therapeutic agent in combination with cytotoxic drugs in human cancer therapy.

## 5. Conclusions

Low-dose quercetin treatment (1 µM) decreases the expression of migration markers in UM cells, which may reduce their capacity to induce metastases. Our results support that quercetin could be a useful adjuvant agent in the treatment of human metastatic UM.

## Data Availability

The data used to support the findings of this study are included within the article.

## Supplementary Materials

The following supporting information can be downloaded at https://www.mdpi.com/article/10.3390/life15060979/s1. Table S1: List of the primer sequences used for qRT-PCR. Table S2: List of the antibodies used for Western blots.

## Abbreviations

The following abbreviations are used in this manuscript:

MDPI	Multidisciplinary Digital Publishing Institute
DOAJ	Directory of open access journals
TLA	Three letter acronym
LD	Linear dichroism
ABC	ATP-binding cassette
AKT	Protein Kinase B
BAP1	BRCA-1-associated protein 1
BRAF	B-Raf Proto-Oncogene
ccRCC	Clear cell renal cell carcinoma
ECM	Extracellular matrix
ENO2	Enolase 2
GNA11	G Protein Subunit Alpha 11
GNAQ	G Protein Subunit Alpha Q
HO-1	Heme Oxygenase 1
ICAM-1	Intercellular Adhesion Molecule 1
ICR	Institute of Cancer Research
MMP	Matrix metalloproteinase
MALAT1	Metastasis Associated Lung Adenocarcinoma Transcript 1
NF-κB	Nuclear factor-kappa B
pAKT	Phosphorylated Akt
PDAC	Pancreatic ductal adenocarcinoma
PI3K	Phosphatidylinositol 3-Kinase
PI3K/AKT	Phosphatidylinositol 3-Kinase/AKT
PTEN	Phosphatase and Tensin Homolog
ROS	Reactive Oxygen Species
Twist	Twist Family BHLH Transcription Factor 1
UM	Uveal melanoma
