# Supplementary material for: Low-Dose Quercetin Dephosphorylates AKT and Suppresses Proteins Related to Migration in Human Metastatic Uveal Melanoma Cells
Source: Life (Basel). 2025 Jun 18;15(6):979. doi: 10.3390/life15060979 (PMC12193735; doi:10.3390/life15060979)
Supplement: Supplementary file 1 [file life-15-00979-s001.zip › life-3662000-supplementary.pdf]

Table S1. List of the primer sequences used for qRT-PCR.

Primer	Forward	Reverse
**AKT**	5′-AGCGACGTGGCTATTGTGAAG-3′	5′-GCCATCATTCTTGAGGAGGAAGT-3′
**Endoglin**	5′-CGGTGGTCAATATCCTGTCGAG-3′	5′-AGGAAGTGTGGGCTGAGGTAGA-3′
**GAPDH**	5′-TGTAGTTGAGGTCAATGAAGGG -3′	5′-ACATCGCTCAGACACCATG -3′
**HEM (HO-1)**	5′-CCAGGCAGAGAATGCTGAGTTC-3′	5′-AAGACTGGGCTCTCCTTGTTGC-3′
**Maspin**	5′-GGCAATGTCCTCTTCTCTCC-3′	5′-GCCGCTTGATTAGTTTCAGT-3′
**MMP2**	5′-TACTGGATCTACTCAGCCAGCA-3′	5′-CTTCAGGTAATAGGCACCCTTG-3′
**MMP9**	5′-GGGCTTAGATCATTCCTCAGTG-3′	5′-GCCATTCACGTCGTCCTTAT-3′
**PI3K**	5′-AACGAGAACGTGTGCCATTTG-3′	5′-AGAGATTGGCATGCTGTCGAA-3′
**PTEN**	5′-TGGATTCGACTTAGACTTGACCT-3′	5′-GGTGGGTTATGGTCTTCAAAAGG-3′
**Vimentin**	5′-GGGAGAAATTGCAGGAGGAG-3′	5′-AGGTCAAGACGTTGCCAGAGAC-3′

Table S2. List of the antibodies used for Western blots.

Antibody	Origin, catalog number	Dilution applied
**AKT (pan) (11E7) Rabbit mAB**	Cell Signaling #4685S	1:1000
**Phospho-AKT (S473) (D9E) Rabbit mAB**	Cell Signaling #4060S	1:1000
**PI3K p110alpha (C73F8) Rabbit mAB**	Cell Signaling #4249S	1:1000
**PTEN (D4.3) XP(R) Rabbit mAB**	Cell Signaling #9188L	1:1000
**NF-kappaB p65 (D14E12) XP Rabbit mAB**	Cell Signaling, #8242	1:1000
**Rabbit polyclonal antibody to MMP9**	Affinity #AF0220	1:1000
**Rabbit polyclonal antibody to MMP8**	Affinity #AF5446	1:1000
**MMP-2 (D2O4T) Rabbit mAb**	Cell Signaling #87809	1:1000
**Anti-HPRT HPRT1 (P00492) Rabbit mAB**	BOSTER #M00668	1:2000
